# COVID-19: Analysis of Factors Affecting the Economy of Hunan Province Based on the Spatial Econometric Model

**DOI:** 10.3389/fpubh.2021.802197

**Published:** 2022-03-08

**Authors:** Wen-Tsao Pan, Wu Zhonghuan, Chen Shiqi, Xiao Siyi, Tang Yanping, Liang Danying

**Affiliations:** ^1^School of Economics and Management, Hunan University of Science and Engineering, Yongzhou, China; ^2^School of Management, Guangzhou Huashang College, Guangzhou, China; ^3^Institute for Economic and Social Research, Guangzhou Huashang College, Guangzhou, China

**Keywords:** The post-COVID era, spatial econometric model, national economy, spatial autocorrelation analysis, LM test, panel data regression analysis

## Abstract

The COVID-19 pandemic has spread across the country negatively impacting on the economy. This paper uses the panel data of 14 prefecture-level cities from 2015 to 2020 in Hunan to determine the factors and effects of economic downturns based on the spatial econometric model. We calculate the Moran index, so-called the Moran's I, to analyse the impact of each factor on the economy. The results show that the spatial correlation of the cities around Chang-Zhu-Tan is high, and the economic growth of the entire province can be influenced by these cities. These cities should adopt strategies to improve the economy, such as reducing the tax revenues, improving the local financial revenues, and reducing the ineffective educational input. These results can also be helpful for policymakers, who will attempt to retransform the Hunan economy during the post-COVID era.

## Introduction

The COVID-19 pandemic affects economic development. In addition, we found that economic growth rate development in Hunan Province has declined after the outbreak of the epidemic in late 2019. What caused the economic change? How did these changes occur? What are the effects? In this paper, we select 14 prefecture-level cities in Hunan Province as a sample. Based on the spatial econometric model, we analyze the factors influencing the economy of Hunan by using general regression analysis, a spatial lag, a spatial error in MATLAB, we obtain the results of the Moran index or Moran's I, the coefficients of the general regression analysis, and the cluster map; and we further discuss the influence of variables. The variables of imports and exports, the total value of agricultural output, industry, local fiscal revenues, and educational input have led to continuous changes. Due to the epidemic, the amount of import and export trade transactions and the consumption of residents have been seriously reduced and have caused the economy of Hunan to suffer serious losses.

Research on the national economy is diverse, and the scope is quite broad, however, few researchers have studied the national economy based on the spatial econometric model. Elhorst (1, 2) proposed a spatial econometric model generalized equation, which embeds spatial parameter W for the independent and dependent variables and the error term part. In addition to this equation, the spatial lag model (SLM), spatial error model (SEM), spatial Durbin model, and others can be used. He (3) constructs a spatial econometric model to study the effects of economic opening up and international tourism development on urban-rural income and finds that the interaction of the three studied variables has significant spatial correlations, and economic opening up and international tourism development have been effective in reducing the urban-rural income gap, consumption gap, and the bias effect of the two factors on the income gap and consumption gap is significant. Urbanization also has an effect on reducing the urban-rural income gap and consumption. Suyan et al. (4) used the global Moran's I index method to analyze the spatial autocorrelation of water resources to obtain the spatial dependence and spatial heterogeneity of indicators in Shandong Province, added the spatial effect to construct a spatial econometric model, and then analyzed the water use efficiency through LM test. They proposed suggestions to promote the sustainable development of the national economy and society. Bai et al. (5) use panel data from 31 provinces in China from 2007 to 2017. This paper calculates the fertilizer use efficiency (FUE) of agricultural production using the stochastic frontier method and discusses its spatial distribution and characteristics. In addition, the spatial effects of education level, the non-agricultural employment rate, disaster rate, and income of farmers on the FUE were investigated using geographically weighted regression (GWR) model to reveal the spatial dispersion and agglomeration effects of different provinces in 2007, 2010, 2013, 2015, and 2017. In Nuari et al. (6), the multiple linear regression equation of the basic model and error correction model are used to analyze the impact of SME units, the value of SME investment, and the value of SME exports on Indonesia's economic growth. Ehlert (7) explores the relationship between socioeconomic, demographic, and health-related variables and COVID-19-related cases and deaths at the regional level. In addition, significant spillover effects of certain variables on the number of cases in neighbor regions were identified, with indications that differ from the overall impact, leading to further analysis of the regional action mechanism of COVID-19 infection. Undseth et al. (8) explore the issue of long-term sustainability with the economics of space debris by re-examining the economics of space debris and suggesting some action and original perspectives for reducing environmental pollution to policy-makers. Xu and Li (9) studied the relationship between innovative human capital and interprovincial economic growth based on panel data and spatial econometrics. Li et al. (10) measured the equalization of public services and living standards of residents in China based on a spatial econometric model and analyzed the impact of public service equalization on various regions in China. Abate (11) studies the link between macrofluctuations and economic growth from a spatial econometric perspective.

## Methodology

### Data Sources and Index Selection

This paper uses an index of the gross regional product (y) as a dependent variable, and six independent variables were selected: local fiscal revenues (x1), total employee wages (x2), the industrial production index (x3), the total value of imports and exports (x4), educational input (x5), and the total value of agricultural output (x6). This paper analyzes the economy of Hunan Province from various perspectives, such as industry, agriculture, and education, which reflect the degree of influencing factors. The data sources are the Hunan Province information network and Hunan Statistical Yearbook from 2015 to 2019.

### Spatial Correlation Test

The spatial correlation between independent variables and the gross regional product must be tested before spatial econometric analysis can proceed. This is done to show that independent variables are spatially correlated with the gross regional product. The Moran's I index is used to determine the spatial correlation. It is calculated by the following formula:


(1) $$\begin{array}{c} Moran^{{}^{\prime }}I=\frac{\sum_{i=1}^{n}\;\sum_{j=1}^{n}\;W_{ij}\;(x_{i}-\bar{x})(x_{j}-\bar{x})}{S^{2}\;\sum_{i=1}^{n}\;\sum_{j=1}^{n}\;\omega_{\ddot{y}}} \end{array}$$

where, $s^{2}=\frac{1}{n}\sum_{i=1}^{n}{\left(x_{i}-\bar{x}\right)}^{2}\;$, $\bar{x}=\frac{1}{n}\;\sum_{i=1}^{n}x_{i}$, *w*_*ij*_(*i, j* = 1, 2, …, *n*) are the spatial weights of regions *i* and *j*, which are used to represent the spatial relationship between regions *i* and *j*, and *x*_*i*_ and *x*_*j*_ are the observed values of regions *i* and *j*, respectively.

### Spatial Econometric Models

We used commonly spatial econometric models—the spatial lag model (SLM) and the spatial error model (SEM). The SLM measures the spatial correlation of the gross domestic product (GDP) of neighboring regions, and the explanatory variables have spatial spillover effects. The formula for the spatial lag model is as follows.


(2) $$\begin{array}{l} \begin{array}{c} p_{gp_{it}}=\alpha +\text{ρ}WPgdp_{it}+\beta_{1}x_{1}+\beta_{2}x_{2}+\beta_{3}x_{3}+\beta_{4}x_{4} \end{array}\\ \;\begin{array}{c} +\beta_{5}x_{5}+\beta_{6}x_{6}+\varepsilon_{it} \end{array} \end{array}$$

i represents the region; t represents the year; W is the adjacency distance matrix of order n × n; *WPgdp*_*it*_ is the spatial lag term of the explained variable, representing the spatial impact of the variable in one region on the other variable in its neighboring region; ρ is the spatial autoregressive coefficient; α is the constant term; β_*i*_ is the parameter to be estimated for the explanatory and control variables; and ε_*it*_ is the random error vector, here all data are logarithmic treatment is used.

The SEM is expressed as follows:


(3) $$\begin{array}{l} \begin{array}{c} p_{gp_{it}}=\alpha +\text{ ρ}WPgdp_{it}+\beta_{1}x_{1}+\beta_{2}x_{2}+\beta_{3}x_{3}+\beta_{4}x_{4} \end{array}\\ \;\begin{array}{c} +\beta_{5}x_{5}+\beta_{6}x_{6}+\varphi_{it} \end{array} \end{array}$$

(4) $$\begin{array}{c} \varphi_{it}=\text{λ}W\varepsilon_{it}+\mu_{it}\;,\;\mu_{it}\sim N(0,\sigma^{2}I_{n}) \end{array}$$

where ϕ is the random error vector, λ is the spatial error autoregressive coefficient, *Wε*_*it*_ is the spatial lag term of the random error term, and μ is the random error term following a normal distribution.

## Empirical Analysis

### Motivation Analysis

To understand each indicator, we calculate the mean, SD, and coefficient of variation of Hunan's regional GDP for 2015–2020.The results are shown in Table 1. The coefficient of variation is very small, and its value is 0.125523657, which shows that the dispersion of Hunan's regional GDP is very small over these 6 years. Furthermore, we found that Hunan Province's regional gross product grew each year, indicating that national economic development showed an upward trend. Before the outbreak of the epidemic in 2019, the growth rate of the province has reached 8%, and the annual growth rate was faster, but the growth rate took a sharp turn and dropped sharply to only 3.8% until 2020, reaching the lowest level in recent years. This shows that the economy of Hunan Province has been greatly affected by the epidemic, which damaged the national economy. Therefore, this article explores the influencing factors of the national economy.

**Table 1:** Regional GDP of Hunan province in 6 years.

**Year**	**Regional GDP**	**Rate of increase (%)**
2020	41781.49	3.8
2019	39752.1	7.6
2018	36425.78	7.8
2017	34590.6	8
2016	31244.7	7.9
2015	29047.2	8.6

*GDP, gross domestic product*.

*Mean = 35473.645; SD = 4452.7816; Coefficient of variation 0.125523657*.

### Analysis Steps

First, to prove the existence of a spatial correlation between cities and municipalities in Hunan Province with 2019 data, this paper measured the correlation between cities and municipalities in Hunan Province by using GeoDa software to obtain the Moran's I index, local correlation clustering map, and significance map.

Second, to prove that the independent variables have an impact on the gross regional product, we conducted a general regression analysis on the independent variables and the dependent variable with 2019 data. In addition, we need to consider the effects of the spatial error and spatial lag; therefore, we also conducted the regression analysis under the condition of a spatial lag and a spatial error. We can conclude that the SLM has the best accuracy. Therefore, we combined the SLM to analyze the correlation between the independent variable X and the dependent variable Y.

Moreover, to further obtain the positive and negative correlations between the independent and dependent variables with panel data, this paper combined the spatial lagged model and used the MATLAB software to estimate the data from 2015 to 2019 under the joint Ordinary Least Squares (OLS), time and spatial fixed effects, time fixed effects, and spatial fixed effects models. We analyzed the autocorrelation between X and Y by linking time and space. Finally, this paper proposes some strategies for Hunan Province.

### Spatial Local Autocorrelation Analysis

#### Moran Index

**Figure 1:**
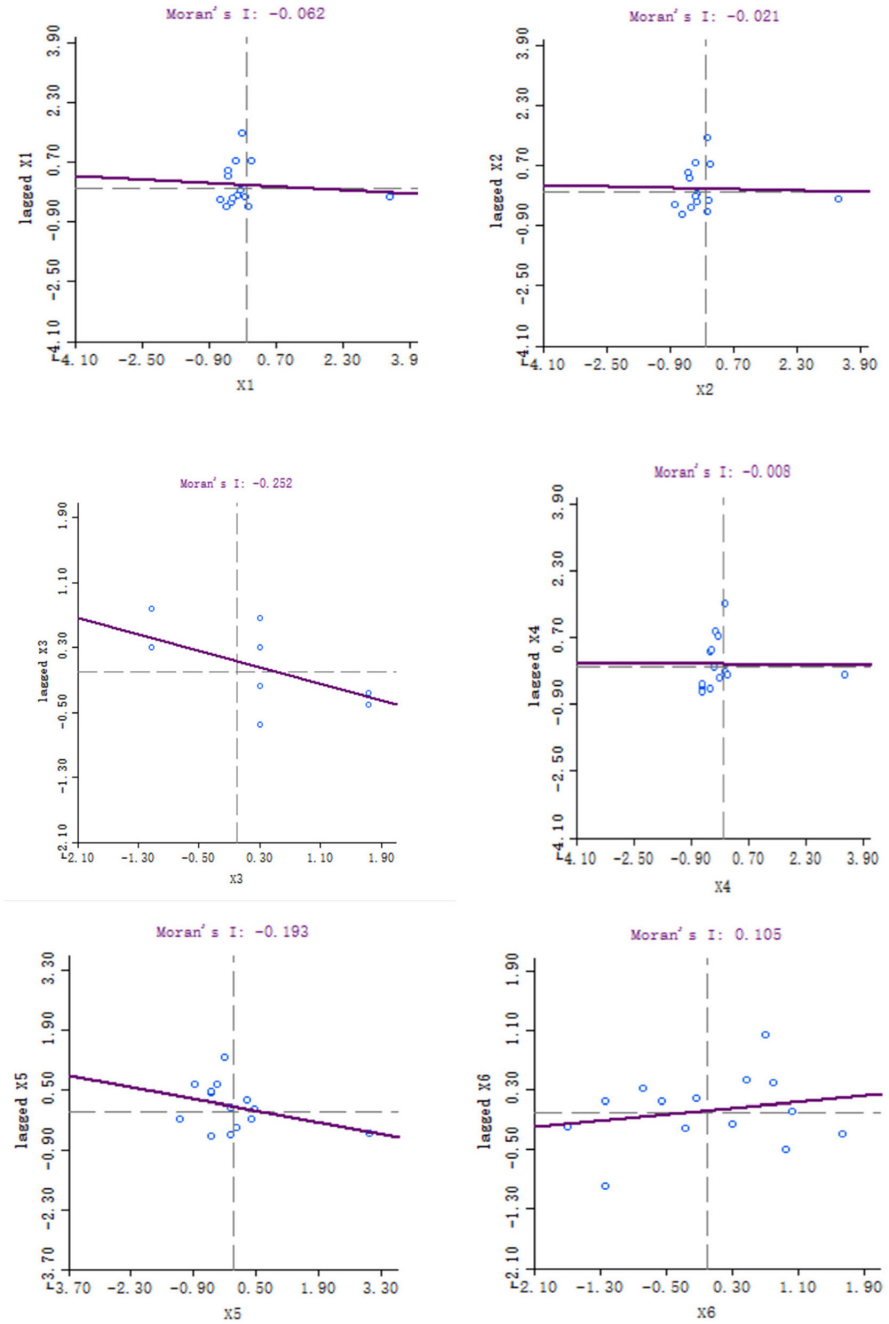
The impact of local fiscal revenue (x1).

The official data of 14 municipalities are input into the GeoDa software to find the local spatial autocorrelation. First, we integrate 2019 data and establish the spatial weight matrix of Queen's collinearity of six variables to obtain a spatial weight file. Then, we used the spatial weight file to draw a scatter plot of Moran's I. The following charts are the output of Moran's I (Figure 1).

The local fiscal revenues (x1), total employee wages (x2), industrial production index (x3), the total value of imports and exports (x4), and educational input of all cities (x5) in Hunan Province are mainly concentrated in the second and fourth quadrants, while the Moran's I is negative, which indicates that the five variables have a negative spatial correlation. The scatter plot of the total value of agricultural output (x6) is mainly distributed in the first and third quadrants, and the Moran's I is positive, which shows that the variable has a positive spatial correlation. The absolute value of the total value of imports and exports is the lowest among the six variables, which shows that this variable has the weakest correlation among adjacent regions. Conversely, the absolute value of education input is the highest, which shows that this variable has the strongest correlation, and the adjacent regions have the greatest influence on each other.

#### Clustering and Significance Analysis

**Figure 2:**
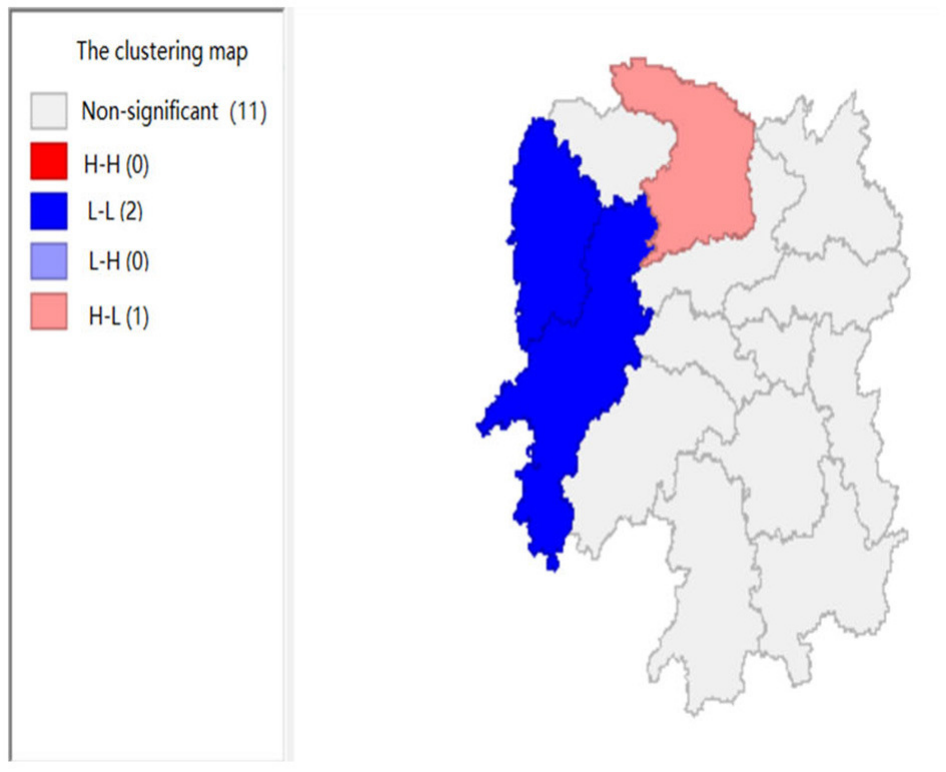
The impact of local fiscal revenue (x1).

**Figure 3:**
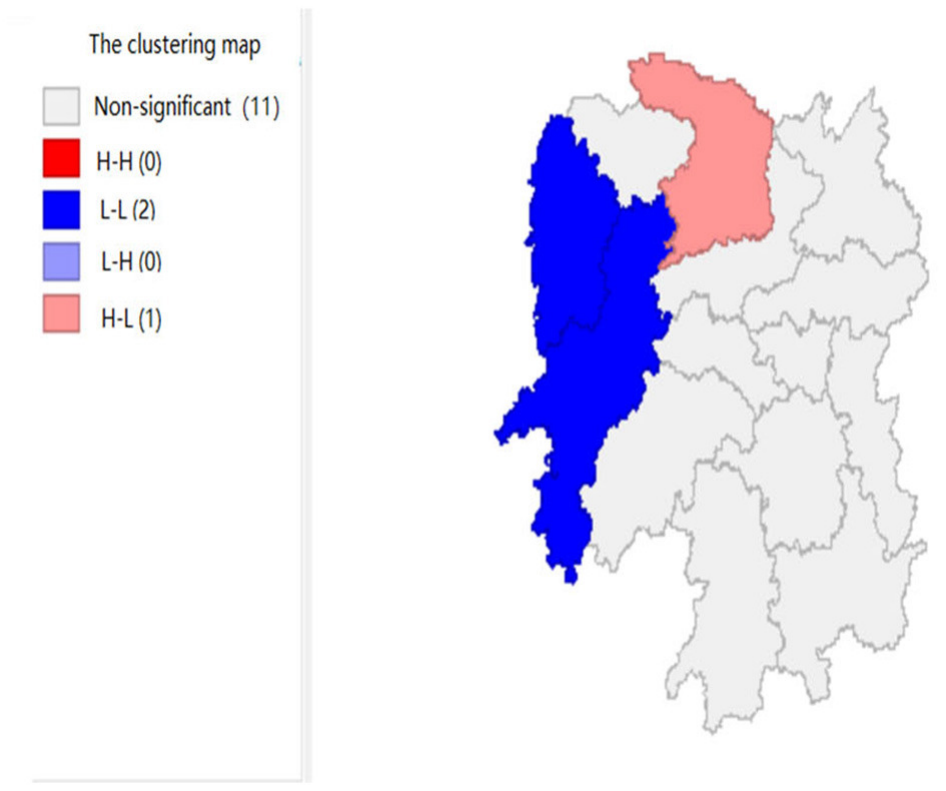
The impact of total wages of employed workers (x2).

**Figure 4:**
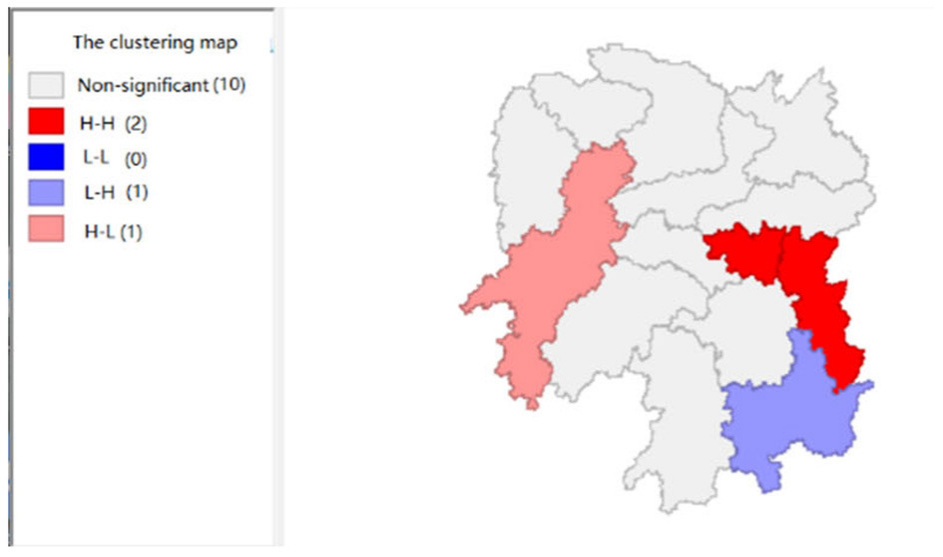
The impact of total value of imported goods (x3).

**Figure 5:**
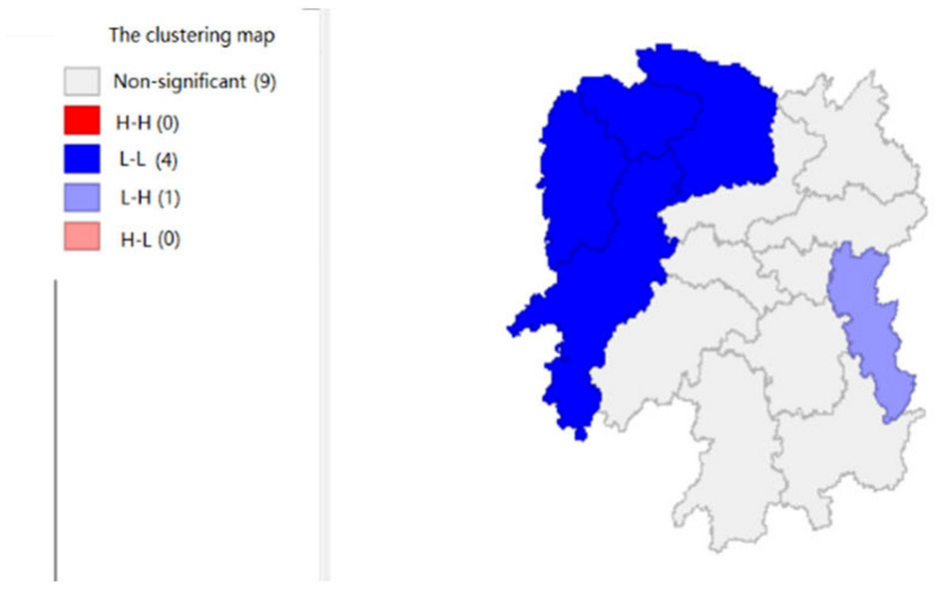
The impact of total value of agricultural output (x4).

**Figure 6:**
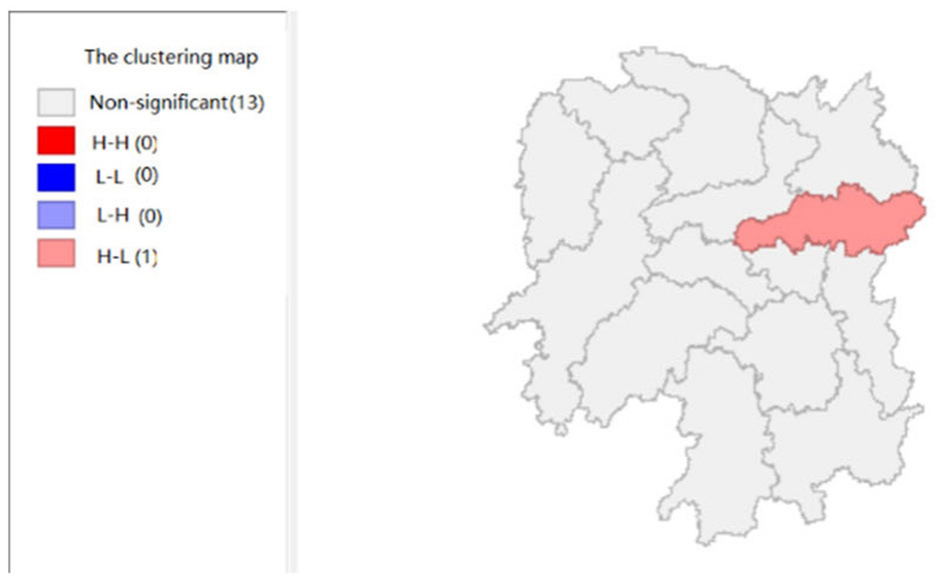
The impact of industrial value-added index (x5).

**Figure 7:**
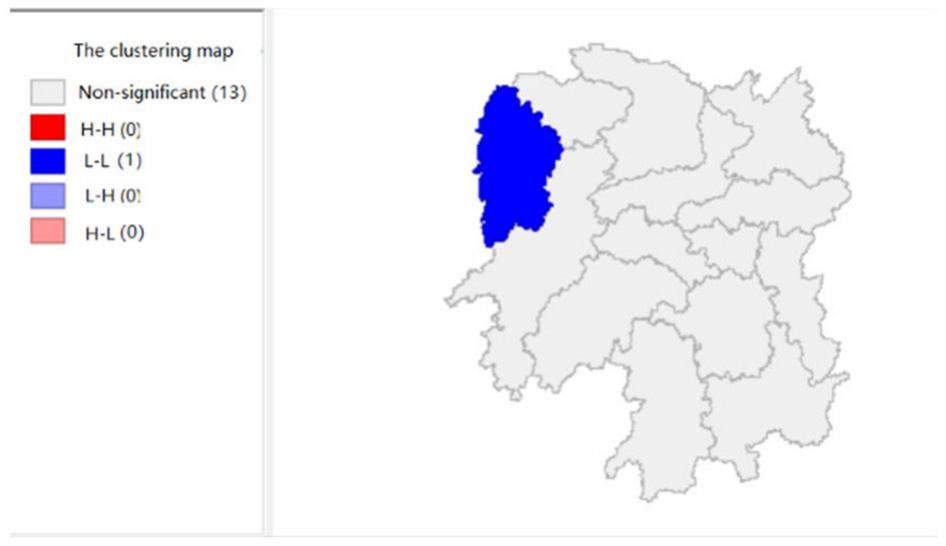
The impact of education input (x5).

**Figure 8:**
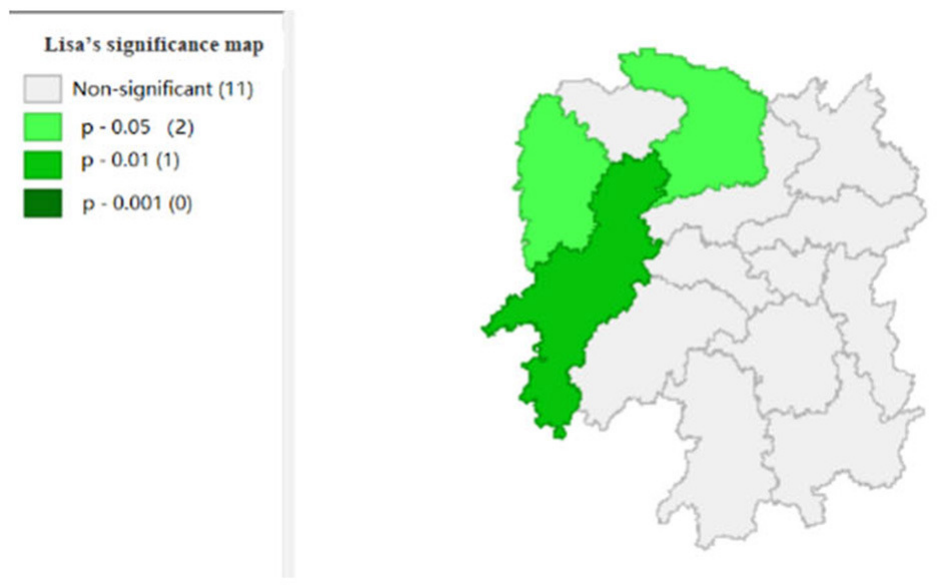
Significance map 1 (x1).

**Figure 9:**
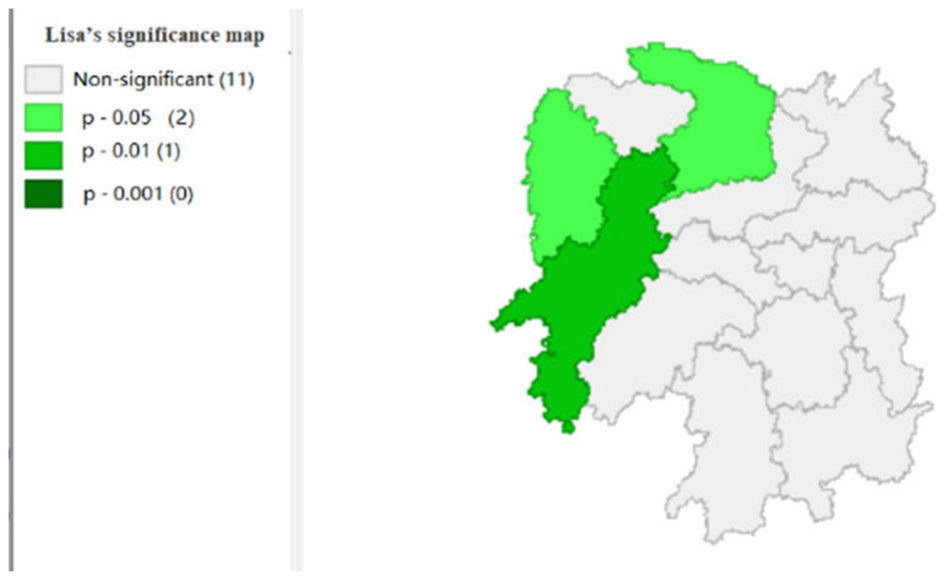
Significance map 2 (x2).

**Figure 10:**
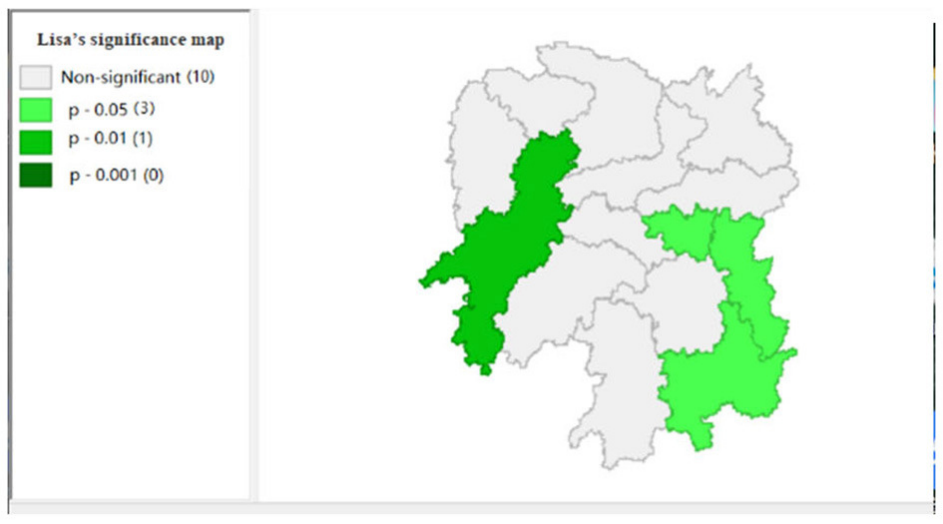
Significance map 3 (x3).

**Figure 11:**
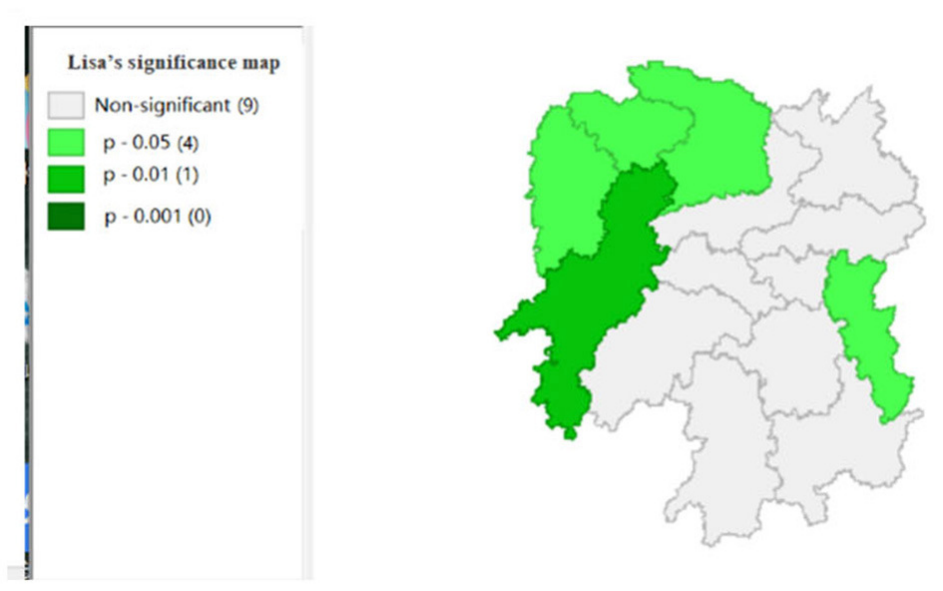
Significance map 4 (x4).

**Figure 12:**
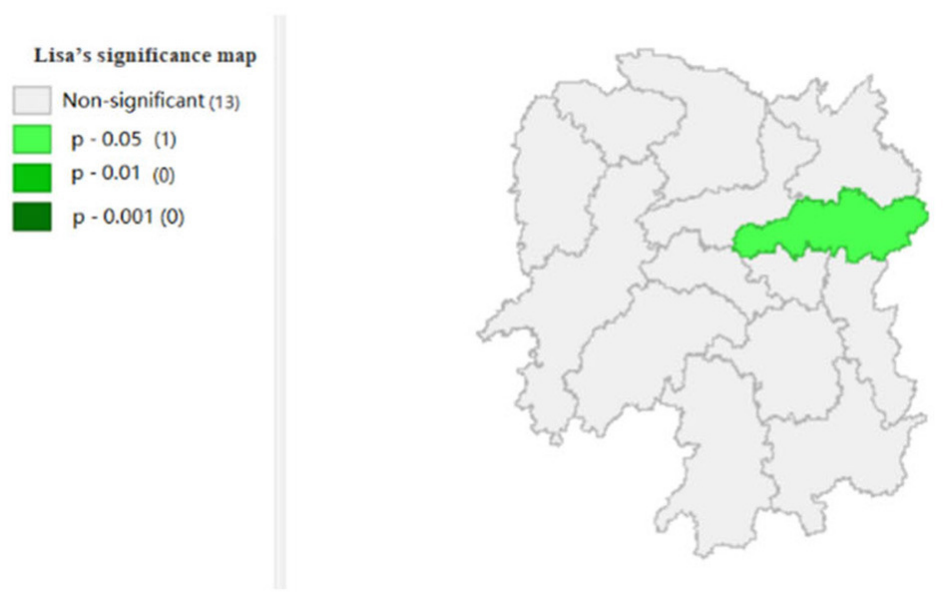
Significance map 5 (x5).

**Figure 13:**
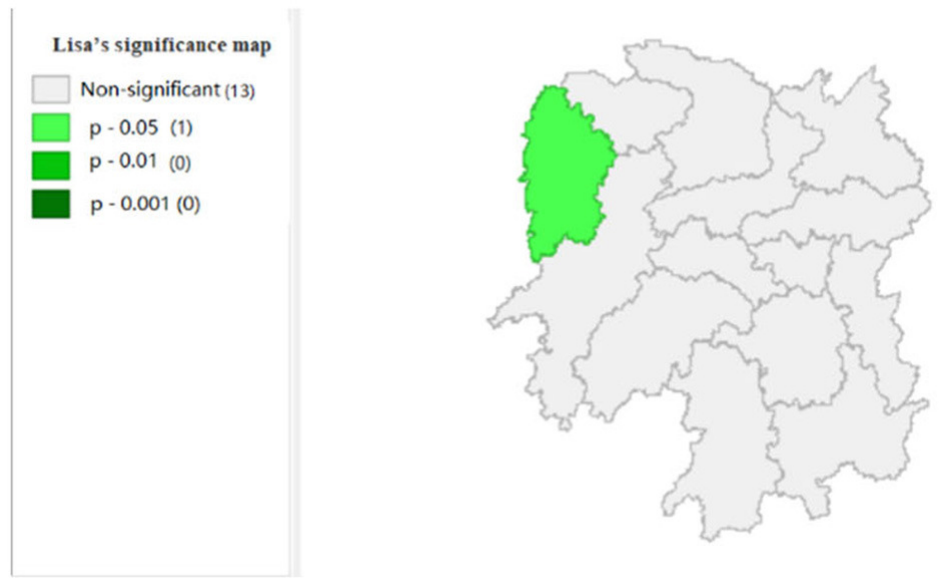
Significance map 6 (x6).

To explore the influence of six factors (the impact of local fiscal revenue, total wages of employed workers, industrial value-added index, total value of imported goods, education input, and total value of agricultural output) on economic development in space in 2019, we use the data to calculate the Moran's I and conduct cluster analysis and the significance analysis of the local spatial correlation, and we obtain the local spatial correlation cluster map and significance map of each city in the year. The cluster map of each variable is shown in Figures 2–7, respectively; and the significance maps are shown in Figures 8–13, respectively.

Figures 2, 3 show that the local fiscal revenues and total employee wages in Hunan Province show obvious “low-low” and “high-low” clustering, indicating that there is a high correlation among Xiangxi, Huaihua, and Changde. From LISA significance maps in Figures 8, 9, the *P*-value of Xiangxi and Changde is 0.05, and the *P*-value of Huaihua city connected with these two cities is 0.01, indicating that Xiangxi, Changde, and Huaihua are highly correlated, and the center is Huaihua. The results showed that the local fiscal revenues and the total employee wages are mutually affected by these cities.

Figure 4 shows that the total value of imported goods in Xiangtan and Zhuzhou cities shows “high-high,” Chenzhou city shows a “low-high,” and Huaihua city shows “high-low,” indicates that the total values of the imported goods from Xiangtan and Zhuzhou are highly correlated and support each other, and Chenzhou is highly correlated with Zhuzhou. However, the total values of imported goods in Chenzhou will be hindered by Xiangtan and Zhuzhou cities. Moreover, the total value of imported goods of Xiangtan, Zhuzhou and Chenzhou also affects Huaihua. If the value of imported goods increases in Huaihua, the total values of these three cities will decrease. In addition, from the LISA significance maps in Figure 10, the *P*-values of Xiangtan, Zhuzhou, and Chenzhou are 0.05, and *P*-value of Huaihua is 0.01, indicating that there is a spatial correlation among the four cities.

Figure 5 shows that the total agricultural outputs of Changde, Zhangjiajie, Xiangxi, and Huaihua present “low-low” clustering and that of Zhuzhou presents “low-high” clustering, showing that Changde, Zhangjiajie, Zhangjiajie, Xiangxi, and Huaihua are relatively concentrated. Furthermore, the agricultural GDP of these four cities negatively affects Zhuzhou. Figure 11 shows that the spatial correlation between Changde, Zhangjiajie, Xiangxi, and Zhuzhou is significant with a *P*-value of 0.05, while the *P*-value of 0.01 in Huaihua reflects the significant impacts of Changde, Zhangjiajie, and Xiangxi on Huaihua.

Figure 6 shows that the industrial value-added index of Changsha exhibits a “high-low” clustering, which indicates that the center of industrial development in Hunan Province is Changsha. Figure 12 shows that the significance of Changsha city is 0.05, which is significant; and there is spatial autocorrelation.

Figure 7 shows that the amount of education input in Xiangxi shows a “low-low” cluster. Figure 13 shows significant performance.

Overall, the local fiscal income, total employee wages, industrial production index, total value of imported goods, education input, and the total value of agricultural output reflect the obvious spatial dual structure characteristics in all cities.

### Regression Analysis

We use the GeoDa Software to analyze 2019 official data. The general regression analysis does not have spatial effects, but if a spatial effect existed in the data, there will be errors in the results (12). Regression analysis is performed under the condition of a spatial error and spatial lag. The results are shown in Tables 2–4. In this paper, we compare the significance of three models to determine which regression model is better and then choose the best model for further analysis.

**Table 2:** Results of general regression analysis.

**Variables**	**Coefficient**	**Std. error**	**t-statistic**	**Probability**
Constant	−21561.9	13305.3	−1.62055	0.14915^−^
X1	−3.97051	4.14586	−0.957704	0.37011^−^
X2	13.1271	3.73844	3.51139	0.00984***
X3	200.884	124.211	1.61727	0.14985^−^
X4	0.000315832	0.000531627	0.594085	0.57115^−^
X5	−17.0679	5.29043	−3.22618	0.01453**
X6	0.000452168	0.000144838	3.12188	0.01680**
R^2^	0.995747	Mean dependent var		2892.86
S.E. of regression	238.42	S.D dependent var		2585.02
Sum squared resid	397,910	Akaike info criterion		197.299
Log likelihood	−91.6496	Schwarz criterion		201.773

In Table 2, the R^2^ is 0.995747, a large value. The results show that the total wages of employees, industrial production index, the total value of imports and exports, and the total value of agricultural output are positively correlated with regional GDP. The combination of the total value of imports and exports and total agricultural output is not significantly correlated with regional GDP, and the correlation between industrial production index and regional GDP is significant. However, the amount of local fiscal revenues and educational input are inversely correlated to regional GDP, and the negative correlation is more significant.

In Table 3, the R^2^ is 0.989472, which shows that the association between the independent variables and dependent variables is strong when using a spatial error regression. It is proven that there is a spatial effect between the independent variables and dependent variables. Moreover, we should consider the correlation between the independent and dependent variables and then conduct regression analysis using the spatial lag. Lambda under the spatial error condition is −0.0816631, indicating that there is a correlation between the independent and dependent variables, suggesting that there is some influence on the economic development among these 14 prefecture-level cities.

**Table 3:** Regression analysis under the condition of a spatial error.

**Variable**	**Coefficient**	**Std. error**	**Z value**	**Probability**
Constant	−9864.87	8738.91	−1.12884	0.25896^−^
X1	−12.6926	4.13371	−3.07052	0.00214***
X2	19.0856	3.96255	4.81648	0.00000***
X3	87.5077	81.4873	1.07388	0.28288^−^
X4	0.000442024	0.000338078	1.30746	0.19106^−^
X5	−6.98233	6.46173	−1.08057	0.27989^−^
X6	0.000230957	0.000188423	1.22574	0.00000***
R^2^	0.989472	Mean dependent var		2892.857143
S.E. of regression	265.245	S.D dependent var		2585.01922
Sigma-square	70354.8	Akaike info criterion		186.414
Log likelihood	−86.206758	Schwarz criterion		190.887

In Table 4, the value-added index, the coefficients of the total employee wage, industrial production index, the total value of imports and exports, and total value of agricultural output are positive, indicating a positive correlation with the regional GDP. Furthermore, the correlation of the total value of imports and exports and regional GDP is not significant, and the correlation of the industrial production index and regional GDP is significant. The coefficients of local fiscal revenues and the amount of educational input are negative, which shows their inverse correlation with the regional GDP; and the negative correlation is significant. Lambda under the spatial lag condition is 2.47702, indicating that the explanatory variables affect each other and that there is a spatial correlation. This indicates that the economic development status among the 14 prefecture-level cities is inconsistent, and we need to propose localized economic measures for the economic development of each city. The R^2^ is 0.996392, which means that the model with spatial lag is best. We will focus on the analysis of variables with a spatial lag and the analysis of the variable data under the spatial lag condition.

**Table 4:** Regression analysis with spatial lag.

**Variable**	**Coefficient**	**Std. error**	**Z value**	**Probability**
Constant	−19,326	8806.79	−1.54529	0.02820**
X1	−8.07057	3.83835	−2.10261	0.03550**
X2	16.7747	3.44211	4.87339	0.00000***
X3	182.772	81.9198	2.23111	0.02567**
X4	0.000515639	0.000368178	1.40052	0.16136^−^
X5	−23.245	5.25555	−4.42295	0.00001***
X6	0.000482438	9.608e−005	5.02121	0.00000***
R^2^	0.996392	Mean dependent var		2892.86
S.E. of regression	155.271	S.D dependent var		2585.02
Sigma-square	24109.2	Akaike info criterion		197.018
Log likelihood	−90.509	Schwarz criterion		202.13

When the *P* < 0.01, the significance is the best and denoted with three asterisks; when the *P* < 0.05, the significance is good with two asterisks; and when the *P* < 0.1, the significance is the worst and denoted with one asterisk. Table 4 shows that the total wages, educational input, and agricultural outputs have three asterisks, indicating that these three variables are very significant and have great impacts on the regional GDP. From the above, we can conclude that the amount of educational input is inversely correlated with regional GDP, i.e., the larger the amount of educational input is, the lower the regional GDP. Hunan municipalities can moderately reduce the amount of educational input. There is a positive correlation between the total wages and the total agricultural output. We suggest increasing total wages to promote consumption and agricultural development.

From the above analysis, we know that the amount of education input is inversely correlated with regional GDP. The larger the amount of education input is, the lower the regional GDP. Therefore, we suggest that Hunan municipalities can moderately reduce the amount of education input. Furthermore, the total wages of employed workers and the total value of agricultural output are positively correlated. We suggest that the total wages should be increased to drive consumption, and at the same time, promote agricultural development.

### Panel Data Analysis

#### LM Test Comparison

To further understand whether there was spatial autocorrelation, the joint OLS, time and spatial fixed effects, time fixed effects, and spatial fixed effects models were applied to data from 2015 to 2019. These models were conducted in MATLAB to diagnose spatial autocorrelation, and the results are shown in Table 5. According to the LM test, the LM spatial lag test of the OLS is 0.0002, which is much smaller than the LM spatial error of 12.3880 ^***^; and the value of the robust LM spatial lag test is also less than the value of the robust LM spatial error test.

**Table 5:** Panel data model for estimating regional GDP with no spatial effect.

**Gross re**		**(1)**	**(2)**	**(3)**	**(4)**
		**Joint OLS**	**Spatial fixed effects**	**Time fixed effects**	**Time and spatial fixed effects**
		0.983	0.987	0.986	0.990
	X1	3.430***	2.907***	2.826***	2.900***
	X2	4.289***	4.391***	4.419***	4.412***
LogP	X3	0.041^−^	−1.007^−^	−1.677*	−0.667^−^
	X4	−0.501^−^	−1.670*	−0.573^−^	−2.013**
	X5	−4.110***	−2.875***	−4.146***	−2.463**
	X6	6.442***	3.580***	6.358***	2.883***
LM spatial lag test		0.0002^−^	0.0000^−^	0.0000^−^	0.0010^−^
LM spatial error test		12.3880***	11.6022***	7.5131***	3.3127*
Robust LM spatial lag test		1.7989^−^	1.3267^−^	0.9845^−^	0.3606^−^
Robust LM spatial error test		14.1866***	12.9289***	8.4977***	3.6724*

*GDP, gross domestic product; OLS, ordinary least squares*.

Regarding the values of the spatial fixed effects, time fixed effects, and time and spatial fixed effects, the values of the LM spatial error test are much larger than the values of the LM spatial lag test, and the values of the robust LM spatial error test are also much larger than the values of the robust LM spatial lag test. This indicates a preference for applying the SEM regardless of the effect model. In the next subsection, we will discuss the results of the SEM.

#### Panel Analysis

In Table 5, the R^2^ for the time and spatial fixed effects is 0.990, which is greater than those the other three models, indicating that the imitative effect in the time and spatial fixed effects model is the best, and the explanatory variables have a greater impact on the explained variable. The values of local fiscal revenues, total employee wages, gross agricultural product, and the amount of educational input in the four fixed effects models receive three asterisks, indicating that the four variables have very significant effects on regional GDP. Local fiscal revenues have a great influence on regional GDP regardless of time, space, or combining both together, and each city region also has an influence on each other even over the long term. In addition, the results of the industrial production index and the total value of imports and exports show that the effect is weakly significant under the four models, so the industrial production index and the total value of imports and exports have little influence on the Hunan economy.

## Conclusion

For the short term, the 2019 data are analyzed in the GeoDa Software. Our suggestions are as follows: (1) as seen in the cluster map and the significance maps, Changde, Zhangjiajie, Xiangxi Tujia, the Miao Autonomous Prefecture, and Zhuzhou have strong spatial correlation and influence on each other. Notably, the indicators of local fiscal revenues and total employee wages are extremely significantly correlated with these cities/states. (2) Improving the local fiscal revenues in Changde, Xiangxi, Zhangjiajie, and Zhuzhou can promote Hunan's economy, increase the revenue of state-owned enterprises, and optimize the expenditure structure to improve economic development. (3) The total values of imports and exports for Xiangtan and Zhuzhou are highly correlated, which means that if the total value of imports and exports in one city is promoted, the total value of imports and exports in the other city will be positively affected. Therefore, to boost regional GDP growth, the foreign trade of neighboring cities should be strengthened, and the prices of imports and exports should be improved. Changsha city has a high correlation with industry, so Changsha should consider developing industry to drive the development of surrounding cities. According to the regression analysis, the amount of educational input can be reduced to lower local fiscal revenue and develop the industry.

For the long term, the data of Hunan Province from 2015 to 2019 are analyzed in MATLAB. According to the panel data analysis, regardless of time and space, the local fiscal revenues, the total employee wages, the total value of agricultural production, and the educational input have very significant effects on regional GDP, and we give the following suggestions: (1) increase the total employee wages, which will drive consumption in the surrounding cities; (2) reduce tax revenues to stimulate economic growth and increase local fiscal revenues; (3) invest considerably in agriculture; and (4) reduce investment in ineffective education and improve the quality of education.

In general, whether in the short or long term, we should focus on the local fiscal revenues, total agricultural output value, and educational input. If we improve these three variables, the GDP in Hunan will be improved.

## Data Availability Statement

The original contributions presented in the study are included in the article/supplementary material, further inquiries can be directed to the corresponding author/s.

## Author Contributions

The authors confirm contribution to the paper as follows: W-TP: study conception and design. CS: data collection. WZ: analysis and interpretation of results. XS, TY, and LD: draft manuscript reviewed the results and approved preparation. All authors the final version of the manuscript.

## Funding

This research was financially supported by the Scientific Research Special Fund of Guangzhou Huashang College (Grant: 2021HSDS02), the general scientific research project of Guangdong Provincial Department of Education's Innovation and Strengthening School Project, Systematic Research on Inter-professional School Simulation Internship Activities and Student Ability Training in Economics and Management (Grant: 2016WQNCX206), and the Hunan Philosophy and Social Science Foundation Project (Grant: 20YBA121).

## Conflict of Interest

The authors declare that the research was conducted in the absence of any commercial or financial relationships that could be construed as a potential conflict of interest.

## Publisher's Note

All claims expressed in this article are solely those of the authors and do not necessarily represent those of their affiliated organizations, or those of the publisher, the editors and the reviewers. Any product that may be evaluated in this article, or claim that may be made by its manufacturer, is not guaranteed or endorsed by the publisher.

## References

[1] Elhorst JP. Spatial panel data models. In: Fischer MM, Getis A, editors. Handbook of Applied Spatial Analysis: Software Tools, Methods and Applications. Berlin: Springer (2010) p. 377–407.

[2] Elhorst JP. Applied spatial econometrics: raising the bar. Spatial Econ Anal. (2010) 5:9–28. 10.1080/17421770903541772

[3] He J. Economic opening, international tourism development, and urban-rural income gap: an analysis based on spatial econometric model. J Yichun Univ. (2019) 27–32.

[4] Suyan L Aojie Z Yao H. Analysis of influencing factors of water use efficiency in Shandong province based on spatial econometric model. Guide Sci Technol Econ. (2021) 124–5.

[5] Bai X Zhang T Tian S Wang Y. Spatial analysis of factors affecting fertilizer use efficiency in China: an empirical study based on geographical weighted regression model. Environ Sci Pollut Res. (2021) 28:16663–81. 10.1007/s11356-020-12246-1 33389465

[6] Nuari R Rahmadana MF Zen Z. (2020). “Analysis of factors affecting economic growth in the SMEs Sector in Indonesia,” in Advances in Social Science, Education and Humanities Research, The 5th Annual International Seminar on Transformative Education and Educational Leadership (AISTEEL 2020), p. 338–43.

[7] Ehlert A. The socio-economic determinants of COVID-19: a spatial analysis of German county level data. Socioecon Plann Sci. (2021) 78:57–9. 10.1016/j.seps.2021.101083 34007090 PMC8120786

[8] Undseth M Jolly C Olivari M. The economics of space debris in perspective. In: Proc. 8th European Conference on Space Debris. ESA Space Debris Office (2021) p. 14–5.

[9] Xu Y Li A. The relationship between innovative human capital and interprovincial economic growth based on panel data model and spatial econometrics. J Comput Appl Math. (2020) 365:377–427. 10.1016/j.cam.2019.112381

[10] Li B Li T Yu M Chen B. Can equalization of public services narrow the regional disparities in China? A spatial econometrics approach. China Econ Rev. (2017) 44:67–78. 10.1016/j.chieco.2017.03.010

[11] Abate GD. On the link between volatility and growth: a spatial econometrics approach. Spatial Econ Anal. (2015) 11:27–45. 10.1080/17421772.2015.1045021

[12] Pietrzak M. Redefining the modifiable areal unit problem within spatial econometrics, the case of the scale problem. Q J Econ Econ Policy. (2014) 9:111–32. 10.12775/EQUIL.2014.014

